# Identification of HK3 as a Potential Key Biomarker in the Progression of Temporomandibular Joint Osteoarthritis via RNA Sequencing

**DOI:** 10.3390/biology14111492

**Published:** 2025-10-25

**Authors:** Ping Luo, Xueliang Lv, Wanting Wan, Hu Qiao

**Affiliations:** 1 Key Laboratory of Shaanxi Province for Craniofacial Precision Medicine Research, College of Stomatology, Xi’an Jiaotong University, Xi’an 710004, China; xjluoping@xjtu.edu.cn (P.L.); lvxueliang_xjtu@163.com (X.L.); wanwantingkq@xjtu.edu.cn (W.W.); 2 Clinical Research Center of Shaanxi Province for Dental and Maxillofacial Diseases, Xi’an Jiaotong University, Xi’an 710004, China; 3 Department of Orthodontics, College of Stomatology, Xi’an Jiaotong University, Xi’an 710004, China

**Keywords:** temporomandibular joint osteoarthritis, HK3, cartilage, RNA-sequence

## Abstract

**Simple Summary:**

Temporomandibular joint osteoarthritis (TMJOA) is a prevalent degenerative disorder with incompletely elucidated mechanisms. Identifying key biomarkers and pathways driving TMJOA progression is crucial for developing targeted therapies. In a rat TMJOA model induced via intra-articular MIA, RNA-seq analysis identified 257 differentially expressed genes. Network analysis pinpointed the glycolytic enzyme HK3 as a critical driver of disease progression, with qPCR confirming its significant up-regulation in damaged cartilage. Integrating the drug–gene interaction database with molecular-docking simulations, we identified three small-molecule inhibitors (MK-8719, LY3372689, and thiamet-G) with high binding affinity for HK3, predicted to inhibit its catalytic activity. To our knowledge, this study is the first to establish a link between HK3 and TMJOA cartilage degeneration, offering a metabolically relevant biomarker for early diagnosis as well as potential disease-modifying therapeutic candidates.

**Abstract:**

The pathogenesis of temporomandibular joint osteoarthritis (TMJOA) is poorly understood. This study aims to identify key biomarkers involved in TMJOA progression and explore potential therapeutic drugs through transcriptome analysis. A rat TMJOA model was established by bilateral injection of monosodium iodoacetate (MIA) into the TMJ cavities. Model validation was conducted using hematoxylin-eosin (HE) and Safranin O-Fast Green (SO-FG) staining. Differentially expressed genes (DEGs) were identified through RNA sequencing. Key pathways were explored using Kyoto Encyclopedia of Genes and Genomes (KEGG), Gene Ontology (GO), and Reactome pathway analyses. DEGs were clustered using MCODE analysis, and Hexokinase 3 (HK3) was identified as a key gene, which was further validated by qPCR. Potential drugs targeting HK3 were selected using the DGIdb database, and molecular docking was conducted to confirm drug-HK3 binding affinity. The TMJOA model was successfully established. RNA-seq analysis revealed 160 upregulated and 97 downregulated DEGs. KEGG, GO, and Reactome pathways analysis identified dysregulated pathways. The top five clusters of DEGs were identified, with HK3 emerging as the key gene. qPCR validation confirmed upregulated HK3 mRNA expression in TMJOA cartilage compared to the control group. Three drugs (MK8719, LY3372689, and Thiamet-G) targeting HK3 were identified through the Drug-Gene Interaction Database (DGIdb) screening, and molecular docking demonstrated high binding affinity between these drugs and HK3. This study suggests that HK3 may play a role in TMJOA progression and could serve as a potential biomarker for inflammatory progression in TMJOA. Targeting HK3 may offer new diagnostic and therapeutic strategies for TMJOA management.

## 1. Introduction

The temporomandibular joint (TMJ) is a synovial joint essential for mastication, respiration, communication, and facial expression [1]. TMJOA is a common degenerative disorder marked by progressive cartilage destruction, subchondral bone remodeling, and chronic pain, significantly impairing patients’ quality of life [2]. Despite its clinical prevalence, the molecular underpinnings of TMJOA remain incompletely understood, and current treatments are largely palliative with limited efficacy. Thus, identifying key biomarkers and pathways involved in TMJOA progression is crucial for developing targeted therapies.

Recent advances in high-throughput sequencing and bioinformatics have enabled a comprehensive exploration of the genetic alterations and molecular mechanisms driving disease onset and progression [3,4]. Transcriptome analysis provides a holistic approach to deciphering the molecular changes associated with TMJOA, offering insights into critical genes and pathways driving disease advancement [5]. Zhang et al. identified hub genes CTGF, FBN1, FN1, EGFR, and ITGA5 as being linked to human TMJOA cartilage degeneration through high-throughput sequencing [6]. Wu et al. identified the key genes Klrk1, Adipoq, Cryab, and Hspa1b in a unilateral anterior crossbite-induced TMJOA model [7]. While the MIA-induced TMJOA model is widely recognized, RNA-seq has been sparingly used to detect differentially expressed genes (DEGs) in this context. Here, we employed RNA-seq to profile DEGs in a mono-iodoacetate (MIA)-induced rat TMJ-OA model and conducted integrative bioinformatics analyses (KEGG, GO, Reactome) to uncover critical pathways. Notably, HK3 was identified as a potential pivotal regulator in TMJOA progression, with its up-regulation confirmed by qPCR. 

To translate mechanistic insights into therapeutic interventions, we further adopted computational drug repurposing strategies. The Drug–Gene Interaction Database (DGIdb) is a valuable computational tool that aggregates curated drug-gene interactions from diverse sources, facilitating drug repurposing and novel therapy development. Integrating data from PharmGKB, DrugBank, and clinical trial repositories, DGIdb enables rapid screening of FDA-approved and investigational compounds with known gene-modulating effects [8]. This approach is advantageous for TMJOA, where traditional drug discovery is hampered by the disease’s complex etiology and lack of validated molecular targets. Drug repurposing can mitigate development risks, shorten timelines, and reduce investment costs [9,10]. In this study, we employed DGIdb to prioritize potential therapeutics targeting HK3, which is a newly identified hub gene in TMJOA pathogenesis. 

To assess the binding affinity of candidate drugs to their target genes, we utilized cavity-detection guided blind docking 2 (CB-Dock2), a robust computational tool renowned for its accuracy in predicting ligand-target interactions based on structural data [11]. This method streamlines drug discovery by identifying high-affinity candidates for subsequent experimental validation, offering significant time and cost savings compared to conventional screening approaches [12]. While prior studies have explored TMJOA using RNA-seq technology [13], none have integrated RNA-seq with DGIdb-based drug repurposing and molecular docking. This study bridges this gap by combining these methods, providing new ideas for the clinical diagnosis and treatment of TMJOA.

By integrating transcriptome analysis, bioinformatics, and drug repurposing strategies, we identified key genes, pathways, and potential therapeutic agents, and this innovative approach holds promise for advancing the development of more effective interventions for TMJOA management. 

## 2. Materials and Methods

### 2.1. TMJOA Model Establishment and RNA-Seq Flow

Ten 8-week-old Sprague Dawley (SD) male rats were sourced from the Laboratory Animal Center at Xi’an Jiaotong University. All procedures were approved by the Institutional Animal Care and Use Committee thereof (Protocol No. XJTUAE2024-2351, 14 August 2024). The rats were randomly classified into two groups: a control group (*n* = 5, administered with PBS) and an experimental group (*n* = 5, injected with MIA). The sample size was determined according to a previous study [14], and animals were fed a standard laboratory chow ad libitum. Each TMJ cavity was injected with either 2 mg/50 µL MIA or an equivalent volume of PBS. After two weeks, cartilage samples were harvested from the TMJs. Adverse effects did not occur in any animals. All animal experiments adhered to the ARRIVE guidelines.

TMJ cartilage tissues were collected, and total RNA was isolated using the RNAiso Plus kit (Takara, Shiga, Japan). mRNA enrichment, fragmentation, reverse transcription, library construction, and sequencing on the BGI-T7 platform were performed by Genergy Biotechnology Co., Ltd. (Shanghai, China). For data analysis, the raw data were processed using Skewer v0.2.2, and data quality was assessed using FastQC v0.11.2. The read length was 2 × 150 bp. Clean reads were aligned to the Rattus norvegicus Rnor 6.0 (https://ftp.ensembl.org/pub/release-98/fasta/rattus_norvegicus/dna/, accessed on 20 October 2025) reference genome using STAR 2.5.2b. StringTie 2.2.1 was used for transcript assembly. The R package DESeq2 v1.38.1 was then used to identify DEGs, followed by functional enrichment analysis of the DEGs.

### 2.2. Histological Staining

Hematoxylin and Eosin (HE) staining and Safranin-O and Fast Green (SO-FG) Staining (Servicebio, Wuhan, China) were employed to assess histological and proteoglycan changes in cartilage.

### 2.3. Identifying DEGs in TMJOA Cartilage Using RNA Sequencing

After data normalization, DEGs between MIA-induced TMJOA samples and negative control samples (PBS group) were identified using DESeq2 software v1.38.1. Genes with |log_2_FC|≥1 and *p*-value ≤ 0.05 were considered differentially expressed.

### 2.4. GO Enrichment, KEGG, and Reactome Pathway Analyses

Gene-disease associations were analyzed using DisGeNET (http://www.disgenet.org/, accessed on 20 October 2025) to identify associated diseases. Functional pathways of the genes were explored through KEGG pathway analysis (https://www.genome.jp/kegg/, accessed on 20 October 2025), Reactome enrichment analysis (https://reactome.org/, accessed on 20 October 2025), and GO analysis (https://geneontology.org/, accessed on 20 October 2025). A *p*-value ≤ 0.05 was deemed statistically significant.

### 2.5. Construction of the Protein–Protein Interaction (PPI) Network

To evaluate functional interactions among DEGs, PPI networks were constructed using the STRING tool (http://www.string-db.org, accessed on 20 October 2025) and visualized in Cytoscape (version 3.10.3). The MCODE plug-in 2.0.3 was then applied to further categorize DEGs and identify potential functional modules or subgroups.

### 2.6. DGIdb Database Used for Drug Prediction Targeting Key Genes

The DGIdb database (https://dgidb.org/, accessed on 20 October 2025) was employed to identify potential candidate drugs targeting HK3. The resulting network was visualized in Cytoscape (version 3.10.3).

### 2.7. Molecular Docking Analysis

The 3D structures of MK8719, LY3372689, and Thiamet-G were retrieved from the PubChem database (https://pubchem.ncbi.nlm.nih.gov/, accessed on 20 October 2025). The HK3 protein structure was sourced from UniProt (https://www.uniprot.org/, accessed on 20 October 2025). Molecular docking was performed using the CB-Dock2 online docking server (https://cadd.labshare.cn/cb-dock2/index.php, accessed on 20 October 2025).

### 2.8. qPCR Validation 

Total RNA was extracted from cartilage using RNAiso (Vazyme, Nanjing, China) and reverse-transcribed into cDNA with RT SuperMix for qPCR (Vazyme, Nanjing, China). qPCR was conducted on a PCR system (Bio-Rad, Hercules, CA, USA) using Cham Q Universal SYBR Mix (Vazyme, Nanjing, China) as per the manufacturer’s instructions. Primer sequences are detailed in Supplementary Table S1.

### 2.9. Protein Domains and Functional Motifs of HK3 Identified via InterPro

The protein domains and functional motifs of HK3 were annotated using InterPro (https://www.ebi.ac.uk/interpro/, accessed on 20 October 2025).

### 2.10. Statistical Analysis

Statistical analyses and graphical representations were performed using GraphPad Prism software 9.0. Comparisons between two groups were made using Student’s *t*-test. Data are presented as mean ± SD, with *p*-values < 0.05 considered significant (* *p* < 0.05, ** *p* < 0.01, *** *p* < 0.001, and **** *p* < 0.0001).

## 3. Results

### 3.1. Establishment of the TMJOA Model and Identification of DEGs

**Figure 1:**
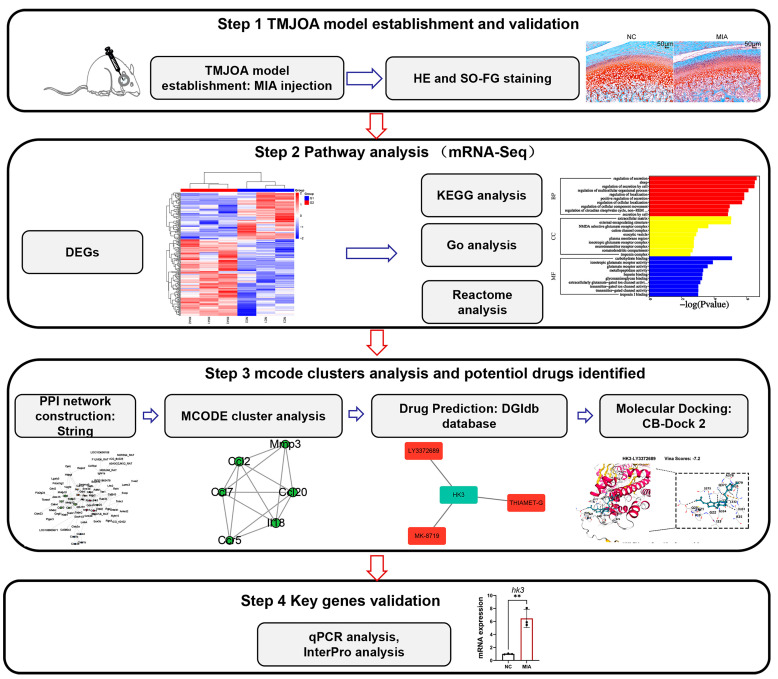
Experimental Flowchart of the Study Design, ** *p* < 0.01.

**Figure 2:**
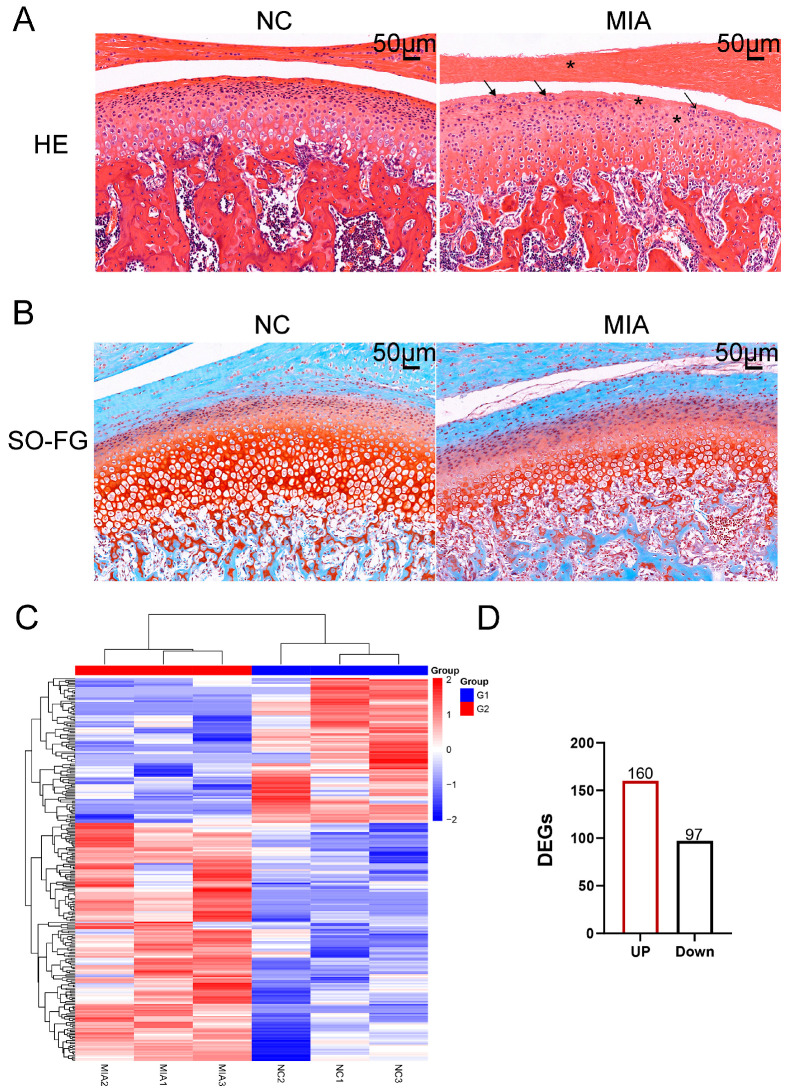
Establishment of the TMJOA Model. (**A**) HE staining. Asterisks represent cell reduction; arrows represent chondrocyte clustering. (**B**) Safranin O and Fast Green staining (SO-FG staining). (**C**) A hierarchical clustering dendrogram was constructed for articular cartilage samples, based on the expression profiles of DEGs. The color scale illustrates normalized expression levels, with red denoting high expression and blue indicating low expression. Each column represents an individual animal, where G1 corresponds to the NC group and G2 to the MIA-induced group. (**D**) Up-regulated genes are marked in red and down-regulated genes in black, and a total of 160 up-regulated and 97 down-regulated transcripts were detected.

The experimental flowchart is depicted in Figure 1. The TMJOA model was induced by intra-articular injection of MIA. H&E staining demonstrated characteristic pathological changes, such as discontinuous articular cartilage surfaces, reduced chondrocyte density in the superficial cartilage layers, chondrocyte clustering, and decreased cellularity in the articular disk (Figure 2A). SO-FG staining revealed a significant reduction in proteoglycan content compared to the NC group (Figure 2B). A heatmap of the DEGs revealed a total of 257 genes with differential expression, comprising 160 upregulated and 97 downregulated genes (Figure 2C,D).

### 3.2. Identification of Key Pathways in the TMJOA Model

**Figure 3:**
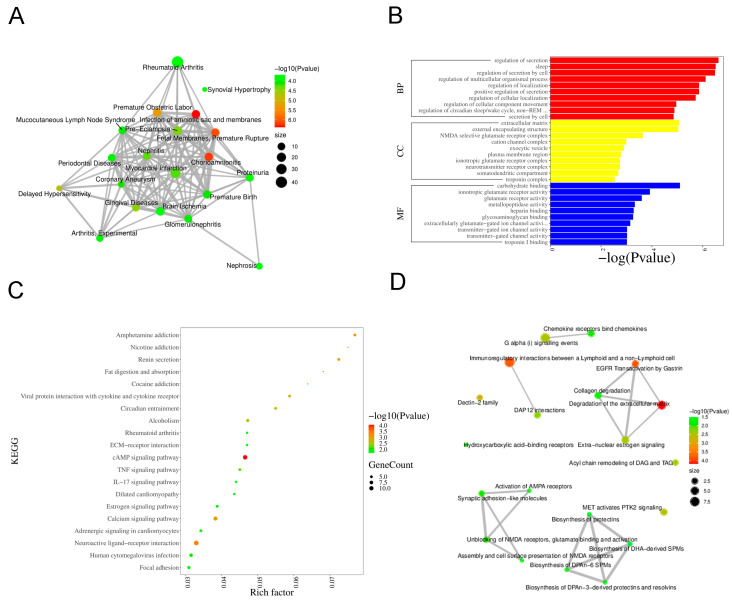
Bioinformatics Analysis Key Pathways in DEGs. (**A**) Based on the sorting information of −Log10 (*p*-value), display the top 20 Diseases. The size of the nodes represents the total number of candidate genes belonging to the Disease. (**B**) GO enrichment analysis. (**C**) KEGG pathway enrichment analysis. (**D**) Reactome pathway analysis.

To explore disease associations of the DEGs, we conducted functional annotation using DisGeNET. Figure 3A illustrates disease enrichment, highlighting significant associations with rheumatoid arthritis, myocardial infarction, and periodontal diseases, suggesting a strong link to inflammatory conditions.

Functional and pathway analyses were performed using KEGG, GO, and Reactome databases. GO enrichment analysis demonstrated that, within the Biological Process category, the most significant terms were “regulation of secretion”, “regulation of localization”, and “regulation of cellular component movement”. The Cellular Component category showed enrichment for “extracellular matrix”, “external encapsulating structure”, and “plasma membrane region”. In the Molecular Function category, “glutamate receptor activity”, “carbohydrate binding”, and “glycosaminoglycan binding” were prominently featured (Figure 3B). KEGG pathway analysis indicated significant enrichment of the “IL-17 signaling pathway”, “TNF signaling pathway”, “ECM–receptor interaction”, and “cAMP signaling pathway” (Figure 3C). Reactome analysis identified key pathways such as “Chemokine receptors bind chemokines”, “Collagen degradation”, and “Activation of AMPA receptors” (Figure 3D).

### 3.3. Identification of Key Clusters 

**Figure 4:**
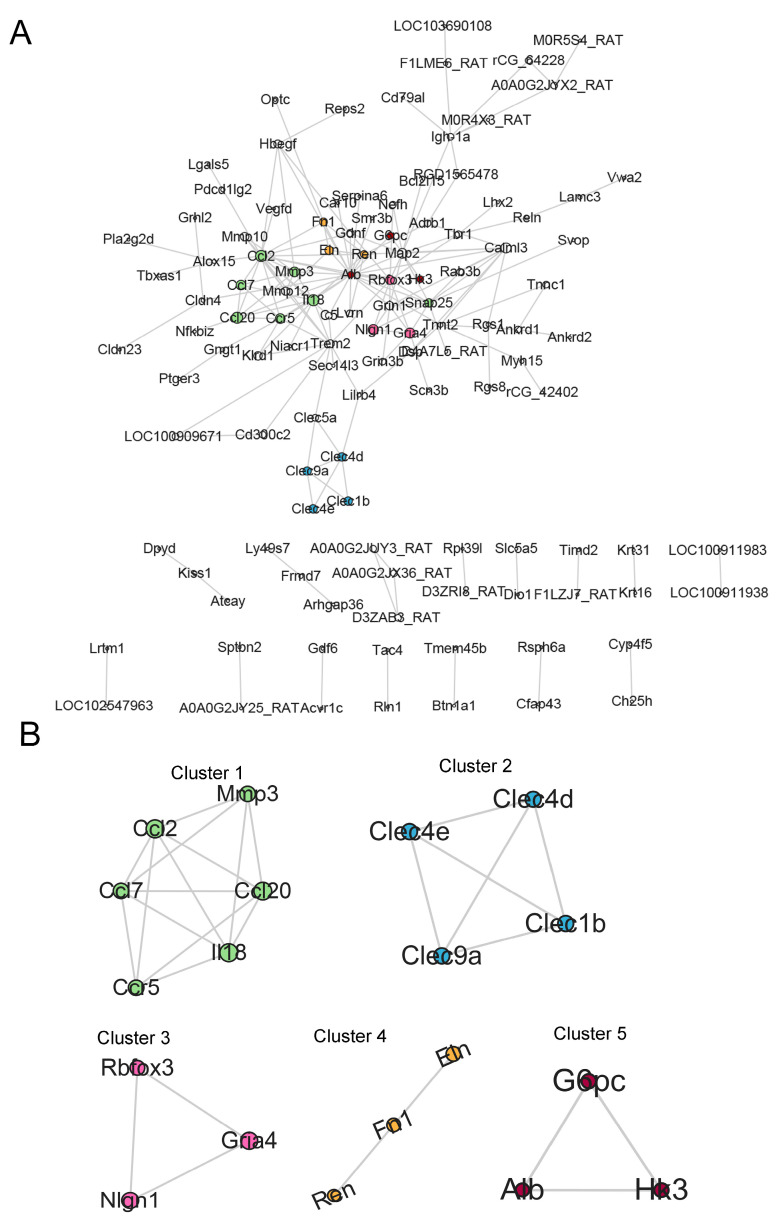
Screening of Hub DEGs. (**A**) PPI network of DEGs. Node size reflects the MCODE score. (**B**) MCODE cluster analysis. The five highest-scoring MCODE clusters (clusters 1–5) are displayed; representative hub genes (e.g., HK3, G6pc, and Alb) are labeled.

The PPI network of 257 DEGs was constructed using the STRING online database (https://string-db.org, accessed on 20 October 2025) with a confidence score threshold of 0.4. The resulting network was then visualized in Cytoscape. Figure 4A displays the 118 most strongly connected proteins. The 118 DEGs were then clustered using MCODE, and the top five clusters of genes are presented in Figure 4B. Cluster 1 was predominantly enriched with immune-related transcripts, including Ccl2, Ccl7, Ccl20, Ccr5, Il18, and Mmp3. Cluster 5 contained metabolic regulators such as Hk3, Alb, and G6pc, while clusters 2–4 comprised genes encoding C-type lectins (Clec1b, Clec4d, Clec9a, Clec4e), glutamate receptors (Gria4), synaptic proteins (Nlgn1, Rbfox3), and extracellular matrix components (Eln, Fn1, Ren).

### 3.4. HK3 Upregulation in Cartilage and Prediction of Protein Domains and Functional Sites via InterPro

**Figure 5:**
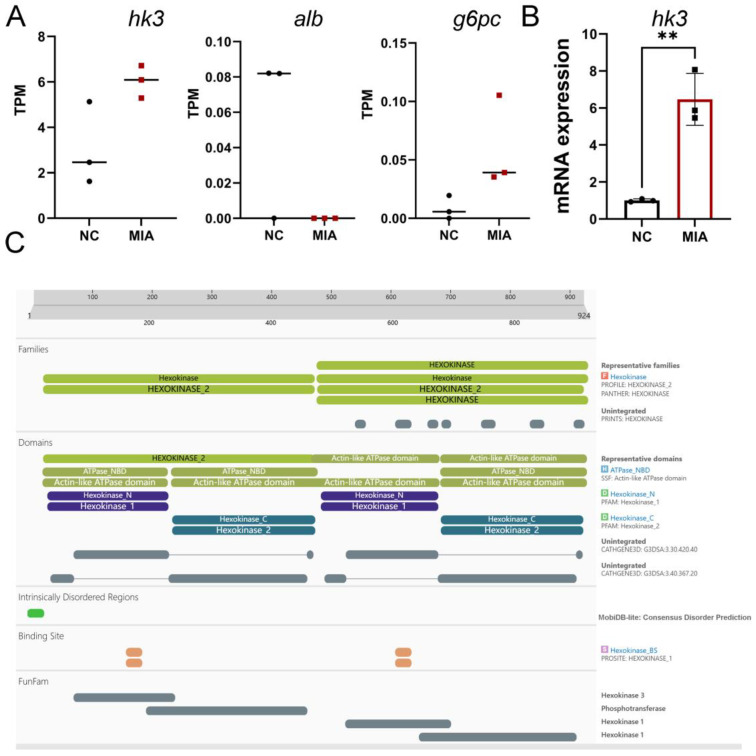
Validation of HK3 as a Candidate Gene. (**A**) TPM values of g6pc, alb, and hk3. (**B**) HK3 mRNA expression quantified by qPCR (*n* = 3), ** *p* < 0.01. (**C**) Predicted protein domains and functional sites of HK3 from InterPro analysis.

Based on key pathway enrichment and existing literature, cluster 5, which is associated with energy metabolism, was selected for further investigation. Given the low expression levels of Alb and G6pc genes and the documented association of HK3 with inflammatory arthritis, HK3 was selected for validation and subsequent experiments (Figure 5A). qPCR analysis confirmed that HK3 mRNA expression was elevated in cartilage (Figure 5B). Furthermore, the protein domains and functional sites of HK3 were predicted using InterPro (Figure 5C). The results indicated that HK3 belongs to the hexokinase family, featuring conserved N- and C-terminal hexokinase domains and associated ATPase modules that facilitate ATP binding and hydrolysis for glucose phosphorylation. Intrinsically disordered regions, substrate/cofactor binding sites, and FunFam annotations provided additional insights into the structural and functional properties of this carbohydrate-metabolizing enzyme.

### 3.5. Exploration of Potential Drugs Targeting HK3

**Figure 6:**
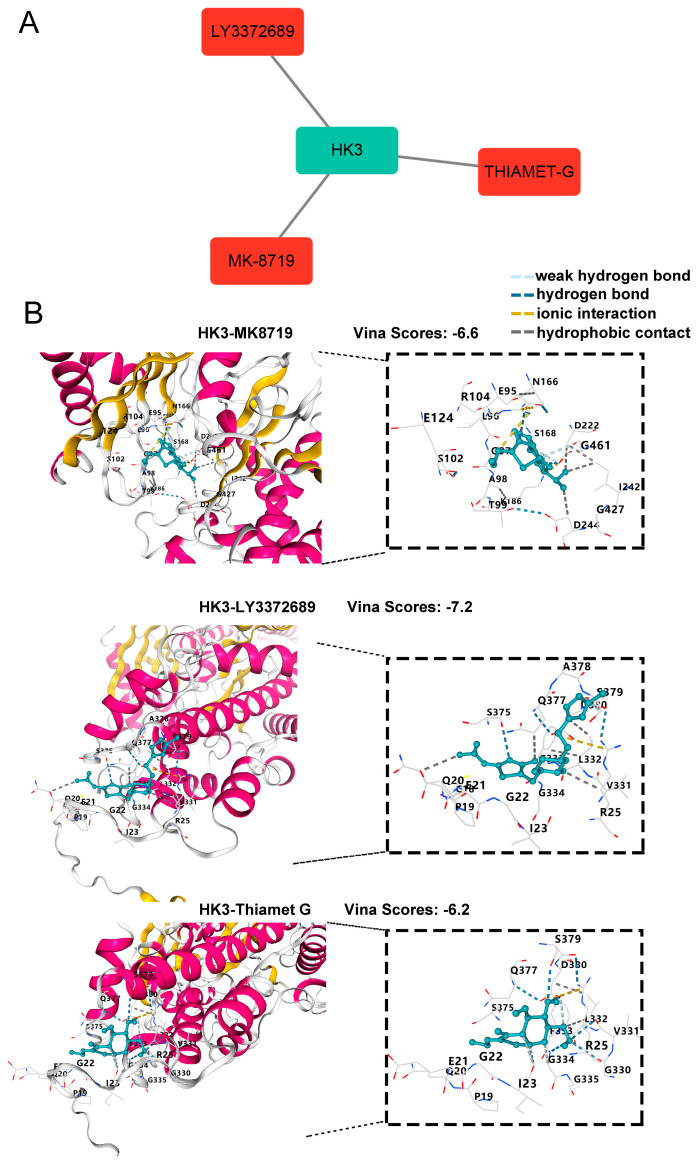
Identification and Validation of HK3-targeting Drugs. (**A**) Candidate drugs targeting HK3 from the Drug-Gene Interaction Database (DGIdb), visualized using Cytoscape. (**B**) Molecular docking simulations were conducted using CB-Dock2. The 3D binding models of MK-8719, LY3372689, and Thiamet-G in complex with HK3 are shown; the pose with the highest Vina score is displayed for each compound. In the interaction diagrams, green dashed lines represent hydrogen bonds, yellow lines denote ionic interactions, and gray lines indicate hydrophobic contacts.

To identify potential drugs targeting HK3, DGIdb was used to explore drug-gene interactions. The analysis revealed three drugs that target HK3: MK8719, LY3372689, and Thiamet-G (Figure 6A). To further evaluate their binding potential, molecular docking was performed using the CB-Dock2 server. Figure 6B illustrates that these drugs exhibit high binding affinity with HK3, suggesting their potential as therapeutic candidates.

## 4. Discussion

TMJOA is a chronic condition marked by cartilage degradation and synovial inflammation, with current treatment options offering limited effectiveness [15]. Despite its prevalence, the precise pathogenesis of TMJOA remains elusive, underscoring the urgent need to identify target genes implicated in its progression and to develop targeted therapeutic strategies. High-throughput sequencing and bioinformatics analysis have become powerful tools for uncovering key genes involved in TMJOA development [16]. Moreover, drug repurposing presents a promising avenue for rapidly identifying suitable medications and aiding in TMJOA treatment [17]. In this study, we employed RNA sequencing of articular cartilage to elucidate the molecular mechanisms underlying TMJOA.

Firstly, the MIA-induced TMJOA model in rats was established, a well-documented approach in multiple studies [18]. HE and SO-FG staining confirmed the successful construction of the model, showing a reduction in surface cells on the articular cartilage and a decrease in proteoglycan content compared to the control group [19]. To pinpoint key pathways and genes involved in TMJOA progression, we conducted gene expression profiling via RNA-seq on cartilage samples collected at 14 days post-induction, identifying 257 DEGs. Subsequent KEGG, GO, and Reactome analyses were performed to further confirm core pathways. The results strongly indicated that the DEGs were predominantly enriched in pathways related to: (i) immune responses (e.g., IL-17 and TNF signaling pathways), (ii) signal transduction (e.g., calcium and estrogen signaling pathways), (iii) extracellular-matrix interactions (e.g., ECM–receptor interaction and collagen degradation), and (iv) metabolic processes (e.g., carbohydrate binding) [20,21]. In conclusion, the RNA-seq analysis of joint cartilage unveiled a complex network of pathways across the GO, KEGG, and Reactome databases. These findings provide a comprehensive overview of the molecular changes occurring in joint cartilage during TMJOA progression and provide valuable insights into the development of future therapeutic interventions targeting the underlying mechanisms of joint disease.

To delve deeper into potential key genes driving TMJOA progression, we employed MCODE clustering and identified five distinct gene clusters. Cluster 1 was strongly associated with inflammation, with IL-18 and MMP3 previously shown to exacerbate TMJOA progression [14,22]. Cluster 2 genes, belonging to the C-type lectin family, are implicated in angiogenesis and inflammation [23]. It has been reported that Clec4e, a member of cluster 2, is expressed in synovial macrophages from human knee OA and shows upregulation under inflammatory conditions [24]. Gria4 in Cluster 3 has been proposed as a biomarker for developing new diagnostic methods for fibromyalgia [25]. Nlgn1 is linked to nerve pain, which might destabilize nociceptive synapses and thus contribute to chronic TMJ-OA pain [26]. Cluster 4 genes were linked to extracellular matrix ECM-related pathways [27,28]. FN1, predominantly localized in the pericellular matrix around chondrocytes and secreted by them, is associated with OA pathophysiology [29]. Cluster 5 contained Alb, G6pc, and Hk3, which are involved in metabolic pathways relevant to inflammation and tissue repair [30]. This enrichment corresponds with the KEGG pathway analysis (Figure 3C), which highlighted the cAMP signaling pathway as significantly altered in TMJOA, emphasizing the pivotal role of metabolic dysregulation in the disease’s pathogenesis. Recent studies suggest that targeting metabolic pathways, as indicated by G6pc and Hk3, could provide novel strategies for managing inflammation and related symptoms in knee arthritis [31]. HK3, which catalyzes the initial, rate-limiting step of glycolysis, is selectively up-regulated in M1-macrophage-driven, pro-inflammatory environments [32]. HK3 was identified as a key protein that is highly expressed in rheumatoid arthritis (RA) synovial fluid. Silencing HK3 curbs lactate-driven histone lactylation, switches macrophage polarity, and dampens synovial fibroblast invasion; consequently, it has been prioritized for mechanistic dissection and therapeutic targeting in RA [33,34]. Given its role in linking glucose metabolism with immune activation, HK3 presents as both a promising biomarker and a viable therapeutic target for inflammatory joint disease, and no studies have explored the role of HK3 in TMJOA. Therefore, we selected HK3 for validation and further experiments, as its key roles in TMJOA remain unexplored and warrant further investigation.

Variations in hub-gene lists across studies may stem from variations in model design or analytic algorithms. Qin et al. developed a unilateral anterior crossbite (UAC)-induced TMJOA mouse model and identified Fgf2, Gsk3b, and Ngfr as key hub genes linking angiogenesis to cartilage inflammation [35]. In human synovial tissue, Zhu et al. employed MCODE cluster analysis to reveal an immune-centric hub signature comprising IL1B, IL10, CCL2, CCL5, CXCL1, CXCL10, ICAM1, CSF1, and MMP1 [36]. Collectively, these studies suggest that both cartilage-intrinsic and synovial-immune factors contribute to TMJOA pathogenesis. In this study, we demonstrate that the glycolytic enzyme HK3 may serve as a metabolic hub, integrating synovial inflammation with cartilage destruction.

To identify potential drugs targeting HK3, we utilized the DGIdb database and identified MK8719, LY3372689, and Thiamet-G as potential candidates. MK-8719 and LY3372689 are potent O-GlcNAcase inhibitors with potential applications in treating tauopathies and Alzheimer’s disease [37,38]. Thiamet-G, another O-GlcNAcase inhibitor, has been shown to increase O-GlcNAc-modified proteins and reduce the production of pro-inflammatory cytokines (IL-6 and IL-8) in arthritis [39]. Furthermore, molecular docking studies confirmed high binding affinity between these drugs, HK3, validating their potential as therapeutic agents for TMJOA. However, further research is necessary to determine their therapeutic efficacy in treating TMJOA. 

Despite its contributions, this study has several limitations. First, our RNA-seq analysis was based on only three biological replicates per condition, so the transcriptomic patterns identified here should be viewed as preliminary pending validation by further research. Second, while HK3 mRNA expression was confirmed, HK3 protein levels were not assessed, and proteomic or functional evaluation of HK3 remains to be conducted in future studies. Third, the therapeutic potential of combining Thiamet-G, MK-8719, and LY3372689 with HK3 targeting for TMJOA treatment requires evaluation in future cell-based or animal experiments.

## 5. Conclusions

In conclusion, our study provides a comprehensive analysis of the molecular pathways involved in TMJOA using RNA sequencing. The findings suggest that targeting the glycolytic gene HK3 may offer a novel therapeutic approach for TMJOA, with MK8719, LY3372689, and Thiamet-G emerging being promising therapeutic candidates worthy of further exploration.

## Data Availability

Data will be made available on request.

## Supplementary Materials

The following supporting information can be downloaded at https://www.mdpi.com/article/10.3390/biology14111492/s1: Table S1: Primers for qPCR analysis.
