# Supplementary material for: Identification of HK3 as a Potential Key Biomarker in the Progression of Temporomandibular Joint Osteoarthritis via RNA Sequencing
Source: Biology (Basel). 2025 Oct 25;14(11):1492. doi: 10.3390/biology14111492 (PMC12650248; doi:10.3390/biology14111492)
Supplement: Supplementary file 1 [file biology-14-01492-s001.zip › biology-3918866-supplementary.pdf]

Table S1. Primers for qPCR analysis.

Gene	Primers
HK3	F: TTCCGTGTCCTGTTGGTACG
	R: CTGGAAGTCCACGATGCAGT
β-actin	F: CCACTGCCGCATCCTCTT
	R: GCATCGGAACCGCTCATT
